# School Nurses’ Experiences of Health Promotion for School-Age Asylum Seekers

**DOI:** 10.1177/1059840520978197

**Published:** 2020-12-10

**Authors:** Saija Inkeroinen, Maija Hupli, Minna Stolt

**Affiliations:** 1 Department of Nursing Science, University of Turku, Finland

**Keywords:** asylum seekers, culturally competent care, cultural diversity, health promotion, qualitative research, refugees, school health services, school nursing, transcultural nursing

## Abstract

The number of school-age asylum seekers and refugees worldwide is increasing. Health promotion provided by school nurses can be crucial for the well-being of young asylum seekers, yet research on these nurses’ experiences is limited. This qualitative study aims to describe school nurses’ experiences of providing health promotion to school-age asylum seekers. Semistructured interviews were conducted with 12 school nurses, and inductive content analysis was then used. The results were grouped under the following themes: (1) difficulties in providing health services to school-age asylum seekers, (2) considering the vulnerable circumstances of asylum seekers, (3) the importance of family-centered health promotion, and (4) the importance of time management. School nurses face challenges that stem from individual asylum seekers’ unique circumstances, nursing competency, and the school health care system. To deepen the existing knowledge, further research is needed from the perspective of asylum seekers.

## Background

The number of displaced people in the world is at a record high, and it is expected to grow further as a result of humanitarian crisis and political unrest. An estimated 30–34 million displaced people are under the age of 18 years (The United Nations Commission on Human Rights [UNCHR], 2020). Asylum seekers apply for international protection due to the fear of being persecuted on the basis of their nationality, ethnicity, religion, or political opinion (UNCHR, 1951). Under international human rights law, asylum-seeking children are entitled to protection and health care (The United Nations [UN], 1989). The importance of promoting their health is recognized internationally on the strategic level (World Health Organization, 2019).

Given that school-age asylum seekers have many complex health problems, this group is in great need of health care and health promotion. Asylum-seeking children have more mental health problems than the local population (Ehntholt et al., 2018; Hanes et al., 2019; Jensen et al., 2019; Kien et al., 2019; Norredam et al., 2018; Yalın Sapmaz et al., 2017), and they are likely to experience post-traumatic stress disorder (Ehntholt et al., 2018; Jensen et al., 2019; Kien et al., 2019). Other health issues observed among asylum-seeking children include communicable diseases (Hanes et al., 2019; Hirani et al., 2018; Mueller-Hermelink et al., 2018; Perniciaro et al., 2018), noncommunicable diseases (Hanes et al., 2019; Hirani et al., 2018), nutrition and growth concerns (Grammatikopoulou et al., 2019; Hanes et al., 2019; Hirani et al., 2018), physical symptoms (Hanes et al., 2019; Hirani et al., 2018), sexual health issues (Hirani et al., 2018; Wikholm et al., 2020), and dental problems (Hanes et al., 2019). The literature supports a clear need for health care.

School nurses are crucial to ensuring that school-age asylum seekers and their families receive health care (Clausson & Cowell, 2019; Johnson et al., 2017; Musliu et al., 2019). They may be the first or only health care professional with whom asylum seekers have a connection in their new country (Clausson & Cowell, 2019; Hansson et al., 2012). School nurses can provide holistic and substantial care for asylum seekers, but to do so requires specific knowledge and a range of skills, and can be mentally demanding (Johnson et al., 2017; Musliu et al., 2019).

Previous studies suggest that school nurses may experience a sense of inadequacy when caring immigrant children and youth. Cultural differences can prevent nurses from acting in their accustomed way (Alizadeh et al., 2011) and require self-awareness (Musliu et al., 2019). Refugees’ and asylum seekers’ traumatic experiences and challenges in acculturation can affect the nurse–child relationship (Musliu et al., 2019; Tellep et al., 2001). School nurses can perceive it as a chance to provide culturally competent care by getting to know other cultures and individual life stories (Musliu et al., 2019; Tellep et al., 2001). Although the importance of school nurses’ role in the care of asylum seekers and refugees has been acknowledged (Clausson & Cowell, 2019; Johnson et al., 2017; Musliu et al., 2019), research into their experiences of providing this care is limited.

Health promotion or health education can be defined as support for students’ self-knowledge and healthy development in a holistic manner; this approach is essential for school nurses (Magnusson et al., 2009; Musliu et al., 2019). Health promotion is especially important when caring for asylum seekers, given that there is a high prevalence of health problems among this group (Farrington, 2020; Johnson et al., 2017; Musliu et al., 2019). Health promotion with asylum-seeking and refugee children should include support to access health care services and recognition of rare health problems, especially relating to past experiences of violence (Lynch, 2001). Health promotion can be challenging for school nurses due to cultural differences, language barriers, and professional-focused approach instead of child-centered care (Magnusson et al., 2009, 2012).

The aim of this study was to describe school nurses’ experiences of the health promotion they provide to school-age asylum seekers (6–17 years). The research aimed to answer the question: What are school nurses’ experiences regarding the health promotion of school-age asylum seekers?

## Method

### Study Design and Sample

Using a descriptive, qualitative study design, semistructured interviews were carried out with school nurses in two cities in southern Finland. Southern Finland was purposively selected for this research because it has the highest population of immigrants in Finland (Official Statistics of Finland, 2019). Contact persons from the study organizations sent information about the study and invitation to participate to school nurses via email. Nurses who were interested to participate in the study contacted the primary researcher by email or phone to negotiate a suitable time and place for an interview. The nurses were eligible to participate in the study if they (1) worked in school health care, (2) had experience of working with school-age asylum seekers in schools, and (3) were volunteering to participate in the study. In Finland, school nurses are public health nurses who have a registered nurse’s degree upon completion of at least 4 years of higher education. Their education includes theoretical studies and practical training. The role of the school nurse is to holistically support, monitor, and care for the health, growth, and well-being of students and the well-being of the school community. The recommended maximum number of students per school nurse is 600, and nurses’ responsibilities can span multiple schools (Poutiainen et al., 2015). School nurses meet every student at least once a year and carry out more extensive health checks at ages 7, 11, and 14 in collaboration with a school physician (Poutiainen et al., 2015). School nurses also collaborate with psychologists, social workers, and other school welfare officials, when needed (Poutiainen et al., 2015). Every minor, including asylum seekers, has the right to access school health care, as set out in international agreements on human rights (UN, 1989) and in national legislation (e.g., The Health Care Act, 2010).

### Data Collection

The interviews were carried out from January to April 2019. The school nurses decided the place and time of their interviews. Most of the interviews were carried out in nurses’ offices, but some were conducted at a nurse’s home, in a library, or in a public meeting place. An interview guide was created based on previous literature (Alizadeh et al., 2011; Hansson et al., 2012; Magnusson et al., 2009, 2012; Tellep et al., 2001) by the research team and refined with peer feedback. The interview guide was piloted with one school nurse: Based on that, no further changes were made. The interviews included questions about nurses’ experiences relating to skills, barriers, opportunities, services, and leadership in school health care and health promotion for school-age asylum seekers, in addition to special characteristics relating to asylum seekers. The main questions (e.g., what are your experiences of working with asylum seekers?) and follow-up questions (e.g., what are the challenges in it?) were asked of all nurses, and amplifying (e.g., Why? How? Could you tell me more?) and confirming questions (e.g., Do you mean xxx?) were presented as the interview proceeded. The interviews were conversational in nature; thus, the nurses could bring up any topic that they perceived to be important. Data collection continued until no new themes were formed, and thus, data saturation was achieved. The interviews lasted from 31 to 80 min, and a total of 619 min of audio recordings were collected.

In every phase of the study, good scientific practice was followed (All European Academies, 2017). The nurses were informed orally and in writing of the purpose of the study, confidentiality, anonymity, voluntary participation, and their right to withdraw from the study. The participating nurses gave written informed consent. Permission to conduct the study was obtained from the departments of health and social services of participating cities, and an ethical approval was granted by the ethics committee of University of Turku (45/2018).

### Data Analysis

The data were analyzed using inductive content analysis (Elo & Kyngäs, 2008). First, the interviews were transcribed, resulting in 201 pages of written data. The researcher became familiar with the data by conducting the interviews, transcribing the recordings, and reading through the written data. Researcher identified and marked sentences or paragraphs answering the research question. The original phrasing was condensed into codes, and these codes were compared and grouped into categories according to their similarities and differences (Table 1). On the basis of these categories, the results were formulated (Table 2; Elo & Kyngäs, 2008). In every phase of the analysis, decisions were discussed and confirmed by the research team. The research team was in frequent contact to evaluate analysis and interpretations. The primary researcher employed a research journal for “reflective commentary” to document the development of analysis (Shenton, 2004). NVivo Version 12 Plus software was used to organize and classify the data (Zamawe, 2015).

**Table 1.** Example of Category Classification During the Data Analysis.

Original Phrasing	Code	Category	Analytical Result
The true need for support is pretty hard to assess. Like, I can assess that you don’t see well, you need glasses, but what kind of support the family truly needs, it’s difficult to evaluate. Because of the language barriers and especially these young adolescents, who are unaccompanied, they have harsh experiences and backgrounds.	The actual need for health services is difficult to assess	Unclear health needs	Difficulties in providing health services for school-age asylum seekers
Issues can be complex or…for example, when a child’s not sleeping well, is it because of a traumatic background or what? It is another problem to define the reason behind it and what could be the proper follow-up unit to look into it.	Complex health problems		

**Table 2.** Categorization of Analytical Results.

Category	Analytical Results
Unclear health needs	Difficulties in providing health services for school-age asylum seekers
Cultural differences	
Lack of mental health competence	
Difficulties in follow-up care	
Health promotion started with the fundamentals	Considering the vulnerable circumstances of asylum seekers
Special issues related to seeking asylum	
Special health content	
Guiding asylum seekers to navigate the local community and health care system	
Families are hard to reach	Importance of family-centered health promotion
Cooperation between school-age asylum seekers, their families, and school nurses is demanding	
Family-centered care is rewarding for school nurses	
Health promotion sessions are time-consuming	Importance of time management
Language barriers	

## Results

### Participants

In total, 12 school nurses participated in the study (out of 89, min. 17 had experience working with asylum seekers). The nurses’ mean age was 47 years (range: 27–57, *SD* = 10.2). They had an average of 21 years of experience in working in nursing (range: 3.5–34, *SD* = 10.4), 14 years of experience as a school nurse (range: 3–31, *SD* = 8), and 5 years of experience in working with school-age asylum seekers in school health care (range: 1–20, *SD* = 5.1). Most of the nurses worked with asylum seekers every week or every month.

### School Nurses’ Experiences of Asylum Seekers’ Health Promotion

The nurses’ experiences concerning the health promotion of asylum seekers in school health care were classified under four themes: (1) difficulties in providing health services to school-age asylum seekers, (2) considering the vulnerable circumstances of asylum seekers, (3) the importance of family-centered health promotion, and (4) the importance of time management (Table 2).

*The difficulties in providing health services to school-age asylum seekers* were related to unclear health promotion needs, cultural differences, lack of mental health competence, and difficulties in providing follow-up care. The complexity of the problems affecting asylum seekers’ well-being due to their traumatic experiences, when combined with language barriers and cultural differences, made it difficult to define health promotion needs or goals. School nurses also needed to be sensitive to cultural differences. They took these differences into account when, for example, discussing sexual health and mental health by explaining that it is natural to talk about them and acknowledging special issues related to asylum seekers’ cultures, such as mental health–related stigma.

…As for traumas, depending on where they (asylum seekers) are from, they don’t identify it, they don’t have such words…They don’t want to see psychologist. If you even say psychologist, they take it wrong, because they think they are crazy. That they will be diagnosed as crazy. (Nurse 12)

However, the nurses felt that they did not have the appropriate skills to care for asylum seekers’ mental health: On occasion, asylum seekers had very serious mental health problems, but the nurses lacked the tools to deal with them and were not sure when to refer an asylum seeker to a mental health specialist.

I want to emphasize that their (asylum seekers) mental well-being worries me. We don’t have the competence and resources…We provide a lot of help and other things, but they need so much more. (Nurse 10)

Moreover, when the nurses identified a need for follow-up care, organizing this was a challenge. The nurses were not familiar with care providers that focused on asylum seekers or what care asylum seekers were eligible to receive.

Issues can be complex or…For example, when child’s not sleeping well, is it because of a traumatic background or what? It is another problem to define what’s the reason behind it and what could be the proper follow-up agency to look into it. (Nurse 2)

*The nurses took into consideration the vulnerable circumstances of asylum seekers* by beginning with the fundamentals, addressing special issues related to asylum seeking, providing tailored health promotion content, and guiding asylum seekers to navigate the local community and the health care system. The fundamentals included education about daily routines, such as the importance of regular mealtimes and personal hygiene.

They are missing preventive thinking that Finnish people have. Importance of breakfast, hygiene, or brushing teeth: We start from the very beginnings. Even with parents, guidance is needed about the importance of breakfast, that it shouldn’t be a mere coffee or something. (Nurse 12)

Not only health promotion but also health care is started with the basics, given that some of the asylum seekers had not received Western medicine in their country of origin and, even if they had, they had no medical records of the care they had received. School nurses did not receive systematically information about children’s status as an asylum seeker. When the nurses knew about it, they were emphatic about the unique situation of people who are seeking asylum, and they wanted to express this to asylum seekers while providing their health promotion. The nurses tried to ensure that asylum seekers experienced fair treatment in school health care and tried to give them hope that they would be granted asylum.

I’m thinking about this one young (asylum seeker)…Lack of resident permit came up. Now we’re waiting for the information this spring. If they can stay or if they have to go back to Iraq. These situations touch my feelings. But I think, somehow, we must inspire hope. The starting point is that they are here and can stay for now. (Nurse 1)

In terms of the content of the health promotion provided, honor-based violence, circumcision, and substance use were important topics to discuss.

Substance use can easily become part of life, if you have hard time here (in Finland), you don’t have much to do, or if school is difficult and you don’t do well in school, or if you can’t sleep…(Nurse 9)

In addition, the nurses felt responsible for guiding asylum seekers to navigate the local community and health care system. They educated asylum seekers and their families about local health and social care services and scheduled appointments with the appropriate authorities. The school nurses had to go beyond their typical role, for example, when providing practical help with booking appointments, pursuing hobbies, and dealing with day-to-day issues.

Fundamentals, like how to take care of child with fever, it’s things like that families need help with. Or how the (health care) system works in Finland or in this city. How do you get to the health care center? This is the kind of guidance they need. (Nurse 1)

*A family-centered approach was experienced by school nurses as essential for the health promotion of asylum seekers*, but families were sometimes hard to reach, and it was necessary to ensure cooperation between school-age asylum seekers, their families, and school nurses. The nurses found the approach demanding yet rewarding. It was difficult for them to contact families by electronic communication, the usual method of communication between school nurses and families in Finland. Some nurses saw this as a sign that the family lacked interest in the child’s life, but others considered that electronic communication was an impractical way of reaching families whose language and technology skills may be limited.

We must send letters. Usually we have events where we teach how to use Vilma (electronic communication software). We try to have parents in school and teach them if we just have a common language. (Nurse 3)

Cooperation between school nurses, asylum seekers, and their families could also be challenging when a large number of family members came to scheduled meetings or when families came to a nurse’s office when they were not expected. The nurses noted imbalances between school-age asylum seekers and their families: Some children ended up in a powerful role in the family due to their faster integration into the local community and better language skills; in other cases, the head of the family dominated the school-age asylum seeker, which made it challenging to be child-centered.

Health promotion sessions…Usually fathers take care a lot of things. So, father has a role, and then there can be the other parent, grandparents, or siblings present too. They come with large number of family members…There can be traumatic experiences, so it is a challenge to make the session’s atmosphere open so that difficult issues can be brought up. (Nurse 8)

Nevertheless, the nurses experienced health promotion with asylum seekers and their families as rewarding. They found it interesting and enjoyed the cross-cultural aspects.

It was new to me, when I came to this school. Many students live nearby. Sometimes the father or, more often, the mother came to my office without an appointment or anything. Like, “It’s easier to talk like this, when you see each other and can explain everything.” Then they say something, like how they tried to make an appointment or something. Somehow, it’s very touching, that they’d rather come to see me. (Nurse 5)

*Time management was experienced as important in the health promotion of asylum seekers* due to time-consuming sessions and language barriers. Given that the nurses began asylum seekers’ health promotion with the fundamentals, they spent a large amount of time trying to obtain medical records, identifying the asylum seeker’s needs, and arranging follow-up appointments. Compared with communication between nurses and Finnish-speaking students, communication between nurses and asylum seekers required much more time, so each could be sure that they understood the other.

It is a challenge, when time is limited, and asylum seekers are group of people who need time. Nothing is going to be done just like that, if we don’t even have a common language. So, taking care of everything requires a lot of time. (Nurse 7)

Professional interpreters were often used, but booking and working with them was time-consuming. Furthermore, some nurses had experienced unprofessionalism from interpreters.

Interpreters are supposed to translate word by word what I’m saying and then translate it back. But I’ve been in situations, where the interpreter and the parent start having conversation with each other, and I’m like “What are you talking about?” . . Sometimes interpreters add their own comments or opinions. The most outrageous (situation) was when interpreter breached professional confidentiality. (Nurse 7)

## Discussion

Knowledge of nurses’ experiences is critical for developing nursing and health care systems that can respond to the growing population of school-age asylum seekers. This study sought to describe nurses’ experiences of providing health promotion to school-age asylum seekers in school health care. Health promotion provided by school nurses is crucial for asylum seekers, but it requires specialized knowledge, skills, and time resources.

The difficulties in providing health promotion were related to health promotion needs, cultural differences, mental health skills, and follow-up care. The nurses experienced difficulties in identifying the health promotion needed. Lack of medical records (Hjern & Kling, 2019; Nakken et al., 2018) and health assessment tools (Hjern & Kling, 2019) are recognized as barriers to identifying the health care needs of school-age asylum seekers and refugees. Needs can also change over time: School nurses have observed higher needs when asylum seekers arrive in or leave a country (Hansson et al., 2012). Asylum seekers have many complex health and well-being needs: Vulnerability due to forced displacement can lead to complex, multidimensional problems (Farrington, 2020; Hjern & Kling, 2019). Some typical school nursing tactics are employed—for example, to identify vaccination needs (Nakken et al., 2018)—but specialized health assessment tools are needed to identify and respond to asylum seekers’ comprehensive health deficits (Hjern & Kling, 2019).

Cultural differences are widely recognized as difficulties for school nurses who are working with asylum seekers and refugees (Alizadeh et al., 2011; Hansson et al., 2012; Musliu et al., 2019; Tellep et al., 2001). When school nurses know and learn about asylum seekers’ cultures, this strengthens the relationship between asylum seeker and nurse (Tellep et al., 2001); thus, cultural education can provide advantages. However, due to the vast number of cultures throughout the world and the many subcultures within cultural communities, school nurses cannot be expected to know about every culture. It is more important to use cultural sensitivity and humility to provide care that is appropriate for asylum seekers’ individual cultures (Hansson et al., 2012; Magnusson et al., 2012). In health promotion, negotiation between asylum seekers and local cultures is crucial (Farrington, 2020; Magnusson et al., 2012; Tellep et al., 2001; Willey et al., 2018). Individualized, child-centered and family-centered care is an essential factor for improving culturally congruent health promotion.

The school nurses in this study were uncertain about their competence in mental health, and they had difficulties when arranging follow-up care. This finding is supported by earlier studies that found that school nurses experienced distress about their insufficient knowledge and skills in addition to the lack of external assistance with children’s mental health (Jönsson et al., 2019; Musliu et al., 2019). Care related to psychological trauma is especially important to school nurses (Musliu et al., 2019). In the context of asylum seekers, emphasis is placed on mental health skills and follow-up care because mental health problems are common in this group. As many as half of all refugee and asylum-seeking children may have psychological problems related to trauma (Jensen et al., 2019; Kien et al., 2019). Nurses acknowledge this and are distressed by the quality of the mental health care they can provide. They had knowledge deficits related to asylum seekers’ health services and care providers, which can prevent asylum seekers from receiving appropriate care. Continuing education about asylum seekers’ care and trauma-related health problems could support nurses’ competence in this area. External care, such as trauma-informed care and psychologist’ services, must be secured to ensure that adequate care is provided for asylum seekers’ mental health.

As for the content of the health promotion provided by school nurses, the fundamentals were perceived to be necessary. This is similar to the experiences of maternity and child health nurses working with asylum-seeking and refugee families (Willey et al., 2018). Vulnerability caused by seeking asylum can lead to extreme changes in person’s hierarchy of needs and thus lead to highlighting the very basic issues of daily living (Farrington, 2020). Asylum seekers have difficulties with navigating local health care systems and have lower health literacy (Chuah et al., 2018; Farrington, 2020; Wångdahl et al., 2015). In this study, the nurses found it rewarding to assist asylum seekers and their families to make use of the local health system, but providing that type of guidance was outside the traditional role of a Finnish school nurse. School nurses were unsure of the eligibility of asylum seekers for health care and need more guidance in this area. As for health literacy, school nurses can improve it (Bjørnsen et al., 2018), yet evidence for their role in supporting the health literacy of asylum seekers and other immigrants is limited (Fernández-Gutiérrez et al., 2018). It would be ideal if school nurses could support empowerment of asylum seekers to navigate the health care system themselves and improve their own health and well-being (Farrington, 2020).

The nurses who participated in this study recognized that honor-based violence and circumcision were important topics of relevance to asylum seekers. This was also found in earlier studies on immigrants in school health care (Alizadeh et al., 2011; Tamaddon et al., 2006). However, the importance of substance use—for example, of alcohol, tobacco, or drugs—is a new finding. The preconception that immigrants make little use of these substances (the “immigrant paradox”) can lead to health care professionals paying little attention to their use in health promotion. Studies support the phenomenon of the immigrant paradox (Harris et al., 2019; Salas-Wright & Vaughn, 2014), yet there seems to be a higher incidence of acute, alcohol intoxications among asylum seekers (Brown et al., 2019). However, research on school-age asylum seekers’ substance use is limited (Horyniak et al., 2016; Posselt et al., 2017). On the basis of our results, school nurses perceive substance use as important; therefore, it should be included in health promotion for school-age asylum seekers.

Reaching the families of asylum seekers for meeting purposes was perceived as a challenge. This result is supported by earlier studies, which found that communication was not effective due to illiteracy or language barriers, lack of transportation, or difficulties in arranging childcare (Tellep et al., 2001; Willey et al., 2018). In our study, school nurses perceived asylum seekers’ technology skills as an additional barrier. Health care in schools should be developed to meet asylum seekers’ expectations and needs. Personal contacts and home visits could be advantageous methods of reaching these families (Tellep et al., 2001; Willey et al., 2018).

Family-centered health promotion is important in school nursing, and it is even more significant when asylum seekers are involved as they may have traumatic experiences affecting family relations. The nurses’ experiences in this study and in earlier studies (Hansson et al., 2012) suggest that families seek health information and other guidance from school nurses. School nurses may be seen as available and easily approachable health care professionals, which is important to asylum seekers and their families. However, some immigrant families perceive the school’s support as insufficient or discriminatory or are fearful of school services (Tulli et al., 2020). This could be due to earlier negative experiences with officials causing mistrust toward health services (Chuah et al., 2018). Thus, there are contradictory findings on families’ experiences of school health care, and this should be studied in depth in the future. Furthermore, not all asylum seekers have family; thus, a shift from the family-centered approach to one that focuses more on the child’s best interests and rights has been suggested (Musliu et al., 2019).

Fundamentals (e.g., education of daily hygiene), language barriers, and the need to use interpreters made providing health promotion sessions for asylum seekers time-consuming. Willey et al. (2018) made the same conclusion in their study with refugee families. Language barriers lead to inadequate explanations, misunderstandings, and provider domination in health promotion provided by school nurses (Hansson et al., 2012; Magnusson et al., 2009, 2012). Language barriers could be overcome by using a professional language interpreter, yet the school nurses mentioned complicated booking systems, time management challenges, and negative experiences of working with interpreters—findings in agreement with earlier studies (Eklöf et al., 2015; Willey et al., 2018). This may explain why language interpreters are not always used, even when there is a clear need for interpretation (Magnusson et al., 2009). With the growing number of asylum seekers, there is a constant demand for interpreters’ services. To respond to this growing need, the competence of professional interpreters should be extended to cover health issues.

### Implications for School Nursing Practice and Research

In summary, promoting health for school-age asylum seekers in school is challenging and time-consuming, and it is provided within the framework of the vulnerable circumstances of asylum seekers and their families. In terms of the implications for clinical practice, school nurses experience rewarding, yet demanding and time-consuming experiences when working with asylum seekers. Nurses’ cultural competency seems to be an essential factor in their success. Cultural competence can be improved by gaining experience of working with multicultural students (Musliu et al., 2019; Tellep et al., 2001) and through continuing education (Govere & Govere, 2016). Health assessment tools for identifying the needs of asylum seekers, in addition to sufficient time resources, should be secured for school nurses who serve asylum seekers. The school health care system should be adapted for asylum seekers by providing a range of methods to reach and assist these families.

This study provided insight into the experiences of school nurses, but future research on school health care must include the perspective of school-age asylum seekers and their families. Furthermore, studies on suitable and effective interventions for asylum seekers in the field of school nursing are warranted. Evaluation of school nurses’ competence and its support could also advance multicultural school nursing, particularly when it comes to mental health competence.

### Trustworthiness of the Study

The trustworthiness of this study is discussed from the perspectives of credibility and transferability (Guba, 1981; Shenton, 2004). In a qualitative study design, credibility is greatly affected by the researcher. The primary researcher has an interest in immigrant health and experience as a school nurse, which deepened the understanding of the phenomenon. However, the researcher’s preconceptions can lead to the risk of bias. To increase the credibility of the study, the research team monitored the process of the study, even though the primary researcher was responsible for the data collection.

The limitations of the transferability of this study are related to the research data. The sample size is small. However, data saturation was achieved. The data may include nurses’ experiences of students who are not asylum seekers. In school health care, there is no system to indicate an asylum seeker’s status; therefore, the difference between asylum seekers, refugees, and other immigrants was not clear to nurses—a phenomenon that has also been recognized in earlier studies (Willey et al., 2018). To improve the transferability of the study, the nurses were encouraged to relate their experiences of students whom they were certain to be asylum seekers. It is also important to note that asylum seekers are not a homogenous group but have individual life experiences and come from numerous different cultures. Thus, the results are to be interpreted at a general level, representing the unique experiences of the participating school nurses. The transferability of the results is also limited by variations from country to country in terms of health services and the job description for school nurses. In Finland and in countries with a similar framework for school nursing (e.g., the Nordic countries), the results can be seen as indicative. A number of school-age asylum seekers are expected to increase globally in the future. Thus, school nurses’ experiences similar to this study can be expected to occur in school nursing internationally.

## Conclusion

School nurses’ experiences of providing health services for school-age asylum seekers are multifaceted. The content and provision of health promotion needs to be tailored to meet the unique needs and expectations of asylum seekers and their families. Nurses acknowledge the challenges arising from asylum seekers’ vulnerable circumstances, the nurses’ competence, and the school health care system’s resources and preparedness for school-age asylum seekers.

## Supplemental Material

Supplemental Material, Supplementary_file_1 - School Nurses’ Experiences of Health Promotion for School-Age Asylum Seekers Click here for additional data file. Supplemental Material, Supplementary_file_1 for School Nurses’ Experiences of Health Promotion for School-Age Asylum Seekers by Saija Inkeroinen, Maija Hupli and Minna Stolt in The Journal of School Nursing
