# Supplementary material for: School Nurses’ Experiences of Health Promotion for School-Age Asylum Seekers
Source: J Sch Nurs. 2020 Dec 10;39(3):229–37. doi: 10.1177/1059840520978197 (PMC10170573; doi:10.1177/1059840520978197)
Supplement: Supplemental Material, Supplementary_file_1 - School Nurses’ Experiences of Health Promotion for School-Age Asylum Seekers [file Supplementary_file_1.pdf]

Supplementary file 1.

Interview guide

Main question	What are your experiences of working with school-age asylum seekers in school health care?
Follow-up questions	Are there any specific features in asylum seekers' care and health promotion?<br><br>Are there challenges in health promotion of asylum seekers?<br><br>Do you have positive experiences in health promotion of asylum seekers?<br><br>What are your experiences of roles of different agencies and instructions in asylum seekers' care?
Main question	What is your preparedness to care for school-age asylum seekers in school health care?
Follow-up questions	What are your strengths in health promotion of asylum seekers?<br><br>What are your weak points in health promotion of asylum seekers?
Example of amplifying questions	Why?<br><br>How?<br><br>Could you tell me more?
Example of confirming questions	Do you mean xxx?

© Saija Inkeroinen, 2019
